# Novel PAMAM-PEG-Peptide Conjugates for siRNA Delivery Targeted to the Transferrin and Epidermal Growth Factor Receptors

**DOI:** 10.3390/jpm8010004

**Published:** 2018-01-09

**Authors:** Koldo Urbiola, Laura Blanco-Fernández, Manfred Ogris, Wolfgang Rödl, Ernst Wagner, Conchita Tros de Ilarduya

**Affiliations:** 1 Department of Pharmacy and Pharmaceutical Technology, School of Pharmacy, University of Navarra, 31080 Pamplona, Spain; kurbiola@alumni.unav.es (K.U.); lblancof@unav.es (L.B.-F.); 2 Department of Pharmaceutical Chemistry, Laboratory of MacroMolecular Cancer Therapeutics (MMCT), University of Vienna, 1010 Vienna, Austria; m.ogris@univie.ac.at; 3 Pharmaceutical Biotechnology, Center for NanoScience (CeNS), Ludwig-Maximilians-University (LMU) 80799 Munich, Germany; wolfgang.roedl@cup.uni-muenchen.de (W.R.); ernst.wagner@cup.uni-muenchen.de (E.W.)

**Keywords:** nanotechnology, cancer, gene therapy, cationic polymers, polyethylenglycol, gene silencing

## Abstract

The transferrin (TfR) and epidermal growth factor receptors (EGFR) are known to be overexpressed on the surface of a wide variety of tumor cells. Therefore, the peptides B6 (TfR specific) and GE11 (targeted to the EGFR) were linked to the PAMAM (polyamidoamine) structure via a polyethylenglycol (PEG) 2 kDa chain with the aim of improving the silencing capacity of the PAMAM-based dendriplexes. The complexes showed an excellent binding capacity to the siRNA with a maximal condensation at nitrogen/phosphate (N/P) 2. The nanoparticles formed exhibited hydrodynamic diameters below 200 nm. The zeta potential was always positive, despite the complexes containing the PEG chain in the structure showing a drop of the values due to the shielding effect. The gene silencing capacity was assayed in HeLa and LS174T cells stably transfected with the eGFPLuc cassette. The dendriplexes containing a specific anti luciferase siRNA, assayed at different N/P ratios, were able to mediate a mean decrease of the luciferase expression values of 14% for HeLa and 20% in LS174T cells, compared to an unspecific siRNA-control. (*p* < 0.05). In all the conditions assayed, dendriplexes resulted to be non-toxic and viability was always above 75%.

## 1. Introduction

During the last years, research has focused on the improvement of non-viral systems for gene therapy. In order to achieve better vectors with enhanced gene delivery activity, different ligands have been included in the formulations. The epidermal growth factor receptor (EGFR) and transferrin receptor (TfR) are two of the most used targeted sites for specific delivery of nucleic acids, since they are overexpressed on the surface of tumoral cells compared with healthy tissues and have demonstrated good activity for drug and gene delivery [1].

The gene transfer capacity of targeted non-viral vectors and novel drug delivery systems including specific ligands has proven to be effective in increasing internalization and delivery of the cargo in vitro and in vivo [2]. However, the inclusion of large proteins in a formulation may result in problems including synthesis procedures and stability; therefore, the necessity of smaller synthetic ligands has increased. The peptides GE11 and B6 do not contain more than 15 amino acids and bind specifically the EGFR and TfR, respectively. B6, described by Xia et al. in a phage display library [3], has been used for targeting l-polyethylenimine (LPEI) polyplexes [4], peptide-polyethylenglycol (PEG)-oligo(ethaneamino)amides [5] and other polymeric systems [6,7]. In a similar way, GE11 peptide was described by Li et al. [8] and subsequently used for the targeting LPEI complexes and other polymers [9,10] with similar results than the ones obtained by EGF protein even in vivo [9]. Concerning the use of these peptides with polyamidoamine (PAMAM) dendrimers, the main objective has pursued the enhancement of the uptake and transfection rate of adenovirus electrostatically coated with PAMAM-G2-PEG-GE11 [11,12,13].

The use of small interfering RNA (siRNA) has become an interesting tool for the specific knockdown of disease-causing genes. A siRNA molecule is constituted by, approximately, 20 pairs of highly specific bases, which are able to activate a complex cellular mechanism that leads to the destruction of a messenger RNA (mRNA). This interaction will have as a consequence the knockdown of the protein coded by the specific mRNA. So far, the use of siRNA in non-viral systems has been based on the use of cationic polymers like polyethyleneimine and chitosan [14,15]. PAMAM dendrimers are considered as an interesting alternative to other cationic polymers due to their low cytotoxicity, controlled molecular weight, high charge density and versatility [16]. Nevertheless, their use as an siRNA delivery system has been limited. Zhou et al. reported the ability of non-degraded PAMAM dendrimers to form nanoparticles in the presence of RNA [17] and other groups have tried to describe the binding mechanism between PAMAM and siRNA, as well as the importance of the nitrogen/phosphate (N/P) ratio, ionic strength of the medium, the size and the dendrimer generation on the complexes [18,19,20]. The activity of this family of molecules has also been studied [21] and, as the presence of targeting is known to improve the activity of non-viral vectors, RNA/dendriplexes have been targeted to the CD44 receptor [22], TfR [23] and EGFR [24]. At the same time, Tsutsumi et al. and Arima and coworkers have extensively studied the behavior of PAMAM dendrimer/α-cyclodextrin conjugates [25,26,27,28,29], and Biswas et al. have improved the behavior of the PAMAM dendrimer by generating a micellar formulation of PAMAM-PEG-DOPE (dioleoylphosphatidylethanolamine)/siRNA complexes [30]. The different strategies adopted for improving the siRNA delivery activity of PAMAM/siRNA complexes has been extensively reviewed elsewhere [31,32].

For the first time, in this work we have evaluated the ability of PAMAM to form nanoparticles in the presence of siRNA, and the gene silencing capacity and cytotoxicity of two new PAMAM conjugates. These novel carriers are coupled to the specific peptides GE11 or B6 via a PEG 2 kDa linker, which have previously been demonstrated to be effective in improving gene delivery in other non-viral systems.

## 2. Materials and Methods

### 2.1. Materials

DMSO (dimethylsulfoxide purissimum), DTT (DL-Dithiothreitol) and TNBS (2,4,6-trinitrobenzenesulfonic acid solution) were obtained from Sigma-Aldrich GmbH (Munich, Germany). Water was used as purified, de-ionized water. NHS-PEG-OPSS (ω-2-pyridyldithio polyethylene glycol α-succinimidylester, 2 kDa) was synthesized by Rapp Polymere GmbH (Tübingen, Germany). Peptides GE11 (CYHWYGYTPQNVI-OH, TFA salt, >95% purity) and B6 (CGHKAKGPRK-OH, TFA salt, >95% purity) by Biosyntan GmbH (Berlin, Germany). LPEI (22 kDa) was synthesized as described by Schaffert et al. [33]. Amine terminated PAMAM dendrimer (Generation 5, 1,4-diaminobutane core, molecular weight 28,854 Da) were purchased from Dendritic Nanotechnologies (Mount Pleasant, MI, 48858, USA). HEPES (4-(2-hydroxyethyl)-1-piperazineethanesulfonic acid) was obtained from Biomol GmbH (Hamburg, Germany). H-l-Cysteine from IRIS Biotech GmbH (Marktredwitz, Germany) and MacroPrep High S from BioRad GmbH (Munich, Germany). Dialysis was performed with spectra/Por membranes (molecular mass cut-off 10 kDa) from Spectrum Laboratories Inc. (Breda, The Netherlands). Cell culture 5× lysis buffer and d-Luciferin sodium salt were purchased from Promega (Mannheim, Germany). Cell culture media, antibiotics, l-alanine-l-glutamine and non-essential aminoacids (NEAA) were obtained from Biochrom (Berlin, Germany). Fetal calf serum was purchased from Gibco (Life Technologies, Carlsbad, CA, USA).

### 2.2. Conjugate Synthesis

#### 2.2.1. Synthesis of PAMAM-PEG(2 kDa)-OPSS

Synthesis of PAMAM-G2-PEG-OPSS was carried out in principle as described recently for LPEI-PEG-OPSS [9] with some modifications. In brief, PAMAM G5 in EtOH was reacted with NHS-PEG-OPSS (2 kDa) dissolved in DMSO for 3 h under agitation at 37 °C. Thereafter, 2 M HEPES pH 7.4, 3 M NaCl and water were added to give a final concentration of 20 mM HEPES and 0.6 M NaCl and pH adjusted to 7.4 using hydrochloric acid. The reaction mixture was loaded on a cation-exchange column (Macro-prep High S; 10/10; BioRad, Munich, Germany) and fractionated with a salt gradient from 0.6 to 3 M NaCl in 20 mM HEPES, pH 7.4. The product eluted between 2 and 2.6 M NaCl and was dialyzed overnight at 4 °C against HBS (20 mM HEPES, 150 mM NaCl, pH 7.4) with a Spectra/Por membrane (molecular mass cut-off 10 kDa). The PAMAM G5 content of the conjugate was determined by TNBS assay, which is used for detection of primary amines. To ascertain the quantity of linker coupled to the PAMAM G5, the amount of dithiopyridine after reduction of an aliquot with DTT was evaluated by spectrophotometric measurement of released pyridine-2-thione (ε_343_ = 8080 M^−1^ × mol^−1^). PAMAM G5-PEG-OPSS was synthesized at a final molar ratio of 1:1.1.

#### 2.2.2. Synthesis of PAMAM-PEG(2 kDa)-OPSS

The peptides GE11 and B6 were added to PAMAM-PEG-OPSS. After 3 h incubation at room temperature, the released thiopyridone was measured at 343 nm to determine the extent of peptide conjugation. Removal of unreacted peptides was carried out on a Macroprep High S cationic-exchange column (Bio-Scale MT 2, BioRad, Munich, Germany) as described previously. The amount of peptide was calculated via the extinction coefficient at 280 nm (ε = 9970 M^−1^ × mol^−1^). The molar ratio of PAMAM G5 to GE11 was 1:0.47 and 1:0.5 for B6.

#### 2.2.3. Synthesis of PAMAM-PEG-Cys

PAMAM-PEG-Cys was synthesized by mixing one part of PAMAM G5-PEG-OPSS with four parts of cysteine at ambient temperature. Purification was carried out on a gel-filtration column (Sephadex G-25; HR10/30 Columns; 20 mM HEPES, pH 7.4) to remove piridine-2-thione and unreacted cysteine. The molar ratio of PAMAM G5 to Cys was 1:1.1.

### 2.3. Preparation of PAMAM-PEG-Peptide/siRNA and LPEI/siRNA Complexes

In general, polyplexes were generated by condensing specific siRNA-luciferase (Luc) or siRNA-Control with PAMAM or PAMAM conjugates at different N/P ratio of PAMAM nitrogen to RNA phosphate. Therefore, PAMAM/siRNA polyplexes were prepared freshly at a final siRNA concentration of 10 μg/mL in HEPES-buffered glucose (HBG 5% (*w*/*w*) glucose, 20 mM HEPES, pH 7.4) by mixing equal volumes of HBG containing the desired amount of siRNA and PAMAM or LPEI. Dendriplexes and polyplexes were allowed to stand for at least 20 min at room temperature before use. More precisely, regarding the DNA nanocomplexation, it should be pointed out that the ability of the polycationic PAMAM polymer to complex the siRNA was previously demonstrated.

### 2.4. Particle Size and Zeta Potential Measurements

The particle size of dendrimer/siRNA formulations and zeta potentials were measured by laser-light scattering using a Zetasizer Nano ZS (Malvern Instruments, Worcestershire, UK). Polyplexes were prepared at a final concentration of 10 µg siRNA/mL in BHG and measured after 30 min of incubation.

### 2.5. Ethidium Bromide Exclusion Assay

A Cary Eclipse spectrophotometer (Varian, Germany) was used for the quantification of ethidium bromide (EtBr) fluorescence at the excitation wavelength λ_ex_ = 510 nm and emission wavelength λ_em_ = 590 nm. 1 mL HBG buffer containing 0.4 μg EtBr was used as blank. After addition of 10 μg siRNA-Control the solution was incubated for 3 min and EtBr fluorescence was assigned to 100%. Increasing amounts of PAMAM or PAMAM-PEG-Peptide corresponding to indicated N/P ratios were added, incubated for 30 s and EtBr fluorescence was determined in relation to the 100% value.

### 2.6. Cell Culture

LS174T/eGFPLuc cell line (LS174T) (colorectal adenocarcinoma) was cultured in RPMI 1640 and HeLa/eGFPLuc (HeLa) (cervix adenocarcinoma) cells were cultured in DMEM. Both cell lines were stably transfected with the eGFPLuc gene cassette as described recently by Su et al. [34]. All cell culture media was supplemented with 10% fetal calf serum, 100 units/mL penicillin, 100 μg/mL streptomycin and 1% l-alanine-l-glutamine (200 mM). All cultured cells were grown at 37 °C in 5% CO_2_ in humidified atmosphere. Cells were passed twice a week by trypsinization.

### 2.7. Gene Silencing Capacity

HeLa and LS174T cells were cultured as described before. For each experiment 10^4^ cells were seeded in 96 well plates 24 h before the treatment. Complexes containing specific siRNA-Luc or siRNA-Control were freshly prepared at the indicated N/P ratios. 20 µL containing 200 ng of siRNA and 80 µL of medium were added to each well and incubated for 4 h. After this time, transfection medium was removed and substituted by 100 µL of fresh culture medium. After 48 h cells were rinsed with PBS and treated with Lysis Buffer (Promega). 30 µL of the lysate were assayed with the Luciferase Assay Kit (100 μL Luciferase Assay buffer, Promega) and a Centro LB 960 plate reader luminometer (Berthold, Bad Wildbach, Germany). The relative light units (RLU) obtained were normalized and presented as the percentage of expression compared to non-treated control cells.

### 2.8. Toxicity Studies

HeLa and LS174T cells were seeded into 96-well plates at a density of 10^4^ cells/well. After 24 h, culture medium was replaced with 80 µL fresh growth medium and 20 µL of transfection complexes. 4 h after, transfection medium was replaced by fresh medium. 48 h post transfection, 10 µL MTT (5 mg/mL) were added to each well reaching a final concentration of 0.5 mg MTT/mL. After an incubation time of 2 h, unreacted dye and medium were removed. The purple formazan product was dissolved in 100 µL DMSO/well and quantified by a microplate reader (Spectrafluor Plus, Tecan Austria GmbH, Grödig, Austria) at a 590 nm with background correction at 630 nm. The relative cell viability (%) compared to control cells containing cells treated with HBG was calculated according to the formula (A_590_ − A_630_) of treated cells × 100/(A_590_ − A_630_) of control cells.

### 2.9. Statistical Analysis

Results are reported as the mean values ± standard deviation. Statistical analysis was performed with SPSS 15.0 (SPSS, Chicago, IL, USA). The different transfection activities in vitro were compared with ANOVA (Tukey post-hoc adjust). Differences were considered statistically significant at *p* < 0.05.

## 3. Results

### 3.1. Size and Zeta Potential Determination

Particle size and surface charge were measured in order to study the influence of the N/P ratio and the presence of the different ligands (Table 1).

Size measurements were performed by dynamic light scattering. As shown in Table 1, particle size was always below 200 nm, presenting an excellent hydrodynamic diameter independently of the N/P ratio used. The presence of the ligand seemed to have little effect on the particle size. Plain and GE11-containing conjugates presented similar sizes and PAMAM-PEG-B6 complexes exhibited the smallest sizes, whereas PAMAM-PEG-Cys presented increased diameters and high polydispersity index (PDI). The other formulations showed PDI values below 0.3.

Surface charge was always positive, with zeta potential values above 20 mV in all the cases. The formulation of the dendriplexes with PAMAM conjugates containing a PEG 2 kDa chain produced nanoparticles with lower zeta potential compared to plain dendriplexes, independently of the N/P ratio used. The presence of the targeting peptide did not influence the zeta potential results.

### 3.2. Ethidium Bromide Exclusion Assay

**Figure 1:**
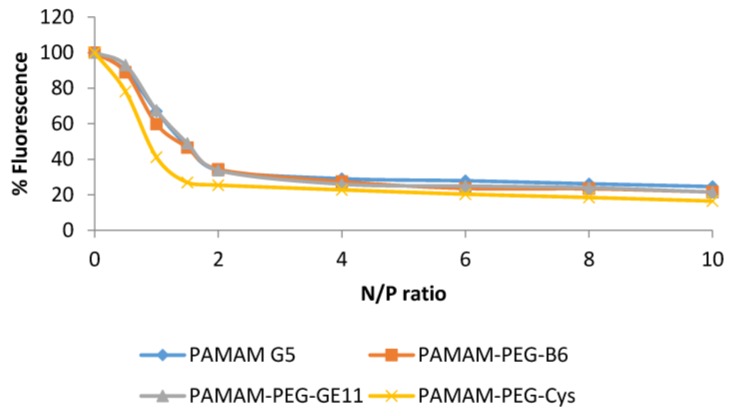
Condensation assay: siRNA condensation measured as a decrease in fluorescence of EtBr added to dendriplexes.

The interaction between PAMAM dendrimers and siRNA was evaluated by the ability of dendrimers to displace the intercalating dye, ethidium bromide, from the siRNA. As shown in Figure 1, as the charge ratio of the complexes increased, the relative fluorescence decreased to a maximum binding degree at N/P ratio 2. The formulation of the dendriplexes with PEG linked peptides did not produce any difference in the behavior of the complexes and similar profiles were obtained.

### 3.3. Toxicity Studies

**Figure 2:**
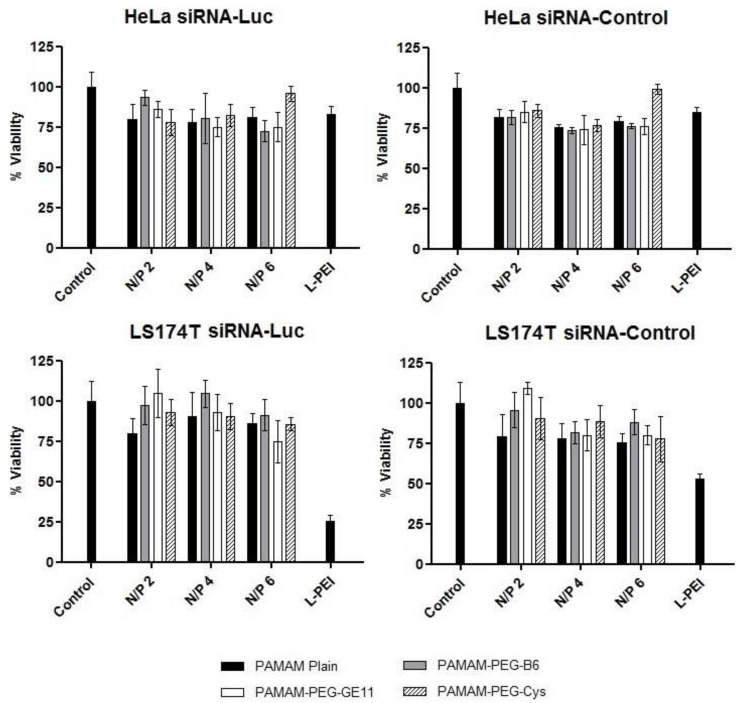
Viability of HeLa and LS174T cells. LPEI (polyethylenimine) polyplexes, PAMAM-plain and targeted PAMAM conjugates with specific siRNA-Luc or siRNA-Control were applied at different N/P ratios. Non-treated cells were included as control. Data are expressed as the mean ± SD (*n* = 4).

For all the dendriplexes tested in this work and for all the N/P ratios, MTT assay was performed (Figure 2). Viability values of dendriplexes were always above 75% in HeLa and LS174T cells. LPEI polyplexes exhibited similar viability values to those obtained for dendriplexes in HeLa, whereas in LS174 cells LPEI treatment lead to a drop of viability values below 60% compared to non-treated cells. Between LPEI polyplexes containing specific siRNA-Luc and siRNA-control in HeLa cells, similar values were obtained; however, in LS174T cells, LPEI polyplexes containing specific siRNA-Luc resulted to be more toxic than PAMAM-dendriplexes, resulting a drop in the viability to 25%.

### 3.4. Gene Silencing Efficacy

In order to explore the blockage of the luciferase expression in two cell lines stably transfected with the cassette eGFPLuc, a specific siRNA (siRNA-Luc) was used in order to block the expression and unspecific siRNA (siRNA-control) was used as a control.

**Figure 3:**
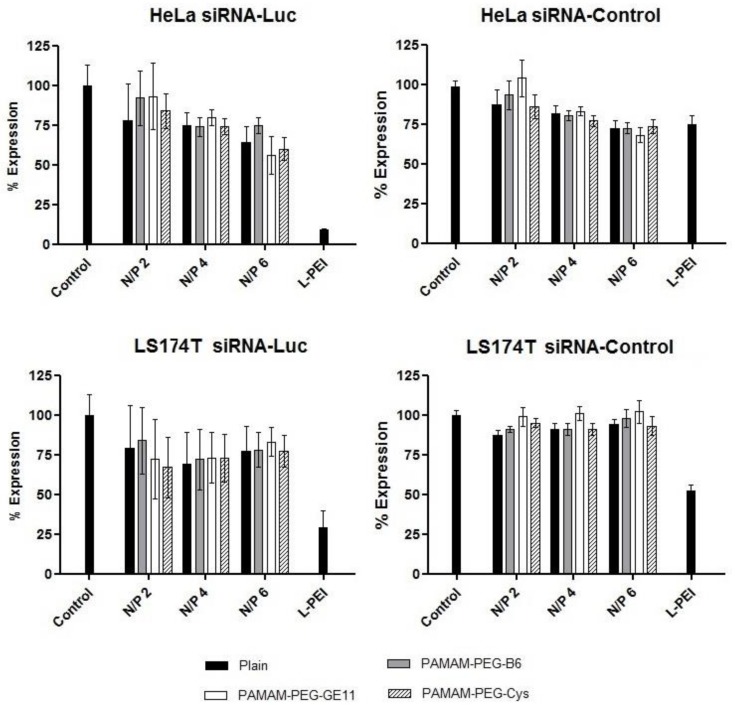
Luciferase silencing efficacy of PAMAM-G5 (Plain) and PAMAM-PEG-GE11/B6/Cys dendriplexes with specific siRNA-Luc or siRNA-control complexes prepared at various N/P ratios. Provided data represent the percentage of expression compared to non-treated (control) cells. Data are expressed as the mean ± SD (*n* = 8). Differences were considered statistically different at *p* < 0.05.

In HeLa cell line (Figure 3), the treatment with siRNA-control produced a decrease in the expression of approximately 20% for all the dendriplexes assayed compared to non-treated cells. The inhibition mediated by the specific siRNA-Luc carrying dendriplexes, compared to control cells, was slightly higher and statistically significant at N/P 4 and 6 ratios. This drop, compared to siRNA-control, represented a mean decrease of 14% of the luciferase expression values (*p* < 0.05). The inclusion of the different ligands was not able to produce differences in any case compared to plain dendriplexes and Cys conjugate containing dendriplexes. LPEI polyplexes carrying specific siRNA-Luc were able to generate a statistically significant decrease in luciferase expression values compared to non-treated cells and the siRNA-Control containing LPEI polyplexes.

Gene silencing efficacy was also assayed in LS174 cell line (Figure 3), where control-siRNA containing dendriplexes produce an average drop of 5% of the luciferase expression values. The mean decrease of the siRNA-Luc containing dendriplexes was around 20% compared to siRNA-control. The inclusion of targeted dendrimers did not produce any difference in the inhibitory activity of the dendriplexes compared to non-targeted ones. LPEI polyplexes were able to mediate an effective decrease of the luciferase values; however, comparing the effect of the specific siRNA-Luc with siRNA-control, the difference was not statistically significant.

## 4. Discussion

One of the main problems to be overcome concerning the use of non-viral systems is the low efficiency and selectivity. The TfR is known to be overexpressed in a variety of tumor cells, as well as the EGFR receptor. Therefore, these receptors are an interesting target for gene therapy improvement. As Scholz et al. claims, the presence of big proteins can lead to problems during synthesis and storage [15]. In order to solve this drawback, the peptides B6 and GE11, specific for the TfR and EGFR respectively, were selected as the targeting moieties to improve the activity of the PAMAM/siRNA dendriplexes.

The size and zeta potential measurements confirmed that the PAMAM dendrimer and PAMAM-PEG-peptide conjugates were able to form nanoparticles with positive surface charge (Table 1). Apparently, significant differences in size could not be described between the plain dendriplexes and the targeted ones, although PAMAM-PEG-B6 containing dendriplexes presented slightly smaller sizes, which could be related with the cationic nature of the B6 ligand. On the other hand, PAMAM-PEG-Cys complexes showed the highest hydrodynamic diameter and PDI. Zeta potential values exhibited a clear drop comparing plain to targeted conjugates. The presence of the linker PEG 2 kDa is likely to be the reason of this variation since the PAMAM-PEG-Cys conjugate containing dendriplexes, which do not contain a peptide in their structure, also present this tendency. In agreement with previous data, the PEG 2 kDa chain produced a decrease in the zeta potential values but did not lead to completely charge neutral particles in LPEI based polyplexes [4,9]. Moreover, it is known that PEG can shield the surface charge of the complexes [35].

One aspect to be considered during the optimization of these kinds of complexes is the importance of the polymer flexibility. In this respect, as siRNA strands contain only up to 20–25 pairs of bases, the structure of the polymer plays an important role during coupling and gene silencing. Kwok et al. studied the stability of LPEI and bPEI siRNA polyplexes, concluding that bPEI showed a better behavior, due to its more flexible branched structure [36]. More flexible PAMAM dendrimers have reported to improve the activity in gene delivery [37]. This approach has been adapted and bigger initial cores of the molecule have been selected with good results [17]. In this study, the PAMAM structure presents a 1,4-diaminobutane instead of the ethylenediamine core used for pDNA delivery previously used in this work, which can provide higher flexibility to the dendrimer. Subsequently, to ensure the formation of a complex, in which the RNA was tightly condensed, an ethidium bromide exclusion assay was performed (Figure 1). The results showed that plain dendriplexes were able to efficiently bind and condense RNA at N/P 2 and higher ratios. The presence of the peptide or the PEG 2 kDa chain did not produce any disturbance of the behavior. These data are in agreement with previous results, which reported that PEG with this molecular weight produces a decrease of the surface charge but it did not negatively influence the DNA condensation of LPEI-PEG-GE11/DNA complexes [9].

The activity of the new conjugates was assayed in modified HeLa and LS174T cell lines stably transfected with the eGFPLuc cassette (Figure 3). The silencing effect of the unspecific siRNA used as control was negligible. When a specific siRNA-Luc was used, the expression values drop by 14% and 20% on average in HeLa and LS174T cells, respectively, compared to the unspecific siRNA-control (*p* < 0.05). The PAMAM-PEG-Cys conjugate did not produce any significant variation of the silencing activity of the complexes compared to the plain dendriplexes and the silencing efficacy was in the same range. Similarly, the conjugation with the specific peptides B6 and GE11 did not produce an improvement of the inhibitory activity of the dendriplexes compared to non-targeted dendriplexes. This lack of improvement, compared to plain dendriplexes, can be partially explained by the presence of PEG, as this molecule is known to reduce the gene delivery activity of different formulations [35,38]. Schafer et al. explored the possibility of targeting LPEI conjugated with the GE11 peptide (LPEI-PEG-GE11/pDNA complexes). In U-87 MG cells, the LPEI-GE11 conjugate was not able to produce higher transfection values compared to non-targeted polyplexes. As they claim, the detargeting can be related with the reduced surface charge or the influence of bulky molecules on the endosomal release [9]. Similar results have been reported by Nie et al. using the peptide B6 as a targeting agent in LPEI-PEG-B6/pDNA based polyplexes. Transfection values in PC3 cells were in a similar range compared to the non-targeted control, nevertheless, the B6 containing polyplexes were able to enhance the cellular association [4]. The siRNA molecule has reached their place of action once in the cytosol and an early release from the endosome is highly desired [15]. The TfR and EGFR-mediated endocytosis are known to imply degradation processes at some extent [39]. At this respect, Martin et al. were able to enhance the transfection activity of B6 peptide-PEG-oligo(ethane-amino)amides only by the presence of chloroquine or other endosomolytic agents [5]. Therefore, if the presence of the PEG chain may block the endosomal release, and a lytic pathway is favored by the presence of the ligand, the proton sponge mechanism of the PAMAM dendrimer structure could not be enough for promoting the rupture of the vesicles and the lack of improvement in gene silencing efficacy could be partially expected.

Despite the fact that the use of these peptides has not been explored with PAMAM dendrimers in gene delivery, Vetter et al. studied the ability of the GE11 peptide to enhance the transfection activity of adenovirus type 5 (Ad) and to achieve a targeted expression in EGFR positive tumors with the binding of Ad to PAMAM-PEG-GE11 conjugates [13]. They compared the PAMAM-G5-PEG-GE11 conjugates with the PAMAM-G2 ones and concluded that PAMAM-G5 was less efficient in terms of targeting compared to G2. The different molecular weight and coupling rate, as well as the necessity of a cooperative binding of several GE11 peptides are the explanations they suggest for this difference [13]. In this respect, the peptide to dendrimer molar ratio should be optimized and new chemical syntheses carried out to modify this ratio. In close relation with the amount of the ligand, the presence of the receptor can play an important role in the gene silencing process. The overexpression of the TfR and EGFR was described in the literature. HeLa cell line has been reported to present high amounts of TfR on its surface [40] as well as EGFR [41,42], similarly LS174T cell line is considered as positive for both receptors [43,44,45]. It is known that the receptor expression varies from one cell batch to another and, hence, differences in the endocytic processes may be expected. Moreover, it is known that each cell line brings a specific behavior with each complex [46]. Because of that, the pr, sence of the specific receptor must be ensured to correlate the behavior of the conjugates.

As expected, the viability values of the targeted complexes were similar to those produced by the non-targeted dendriplexes and were always above 75% (Figure 2). As a consequence, the silencing effect of the treatment with siRNA-Luc dendriplexes must be mediated by the siRNA-Luc specific inhibition. Control LPEI polyplexes resulted to be toxic in LS174T cells, reducing the viability values to 25%, whereas in HeLa cells viability was similar compared to dendriplexes.

The excellent results provided by the toxicity assays make these vectors suitable for further improvement. Moreover, the interesting shielding effect of the PEG chain included can partially explain the lack of improvement in the gene silencing profile, without eradicating the possibility of using this conjugates for in vivo purposes, since the extrapolation from in vitro results to in vivo is still limited for these systems.

## 5. Conclusions

In this work, two novel PAMAM-PEG 2 kDa-peptides conjugates targeted to the TfR and EGFR have been evaluated. The presence of the peptides B6 and GE11 did not produce any variation in the size and zeta potential values, presenting always a nanometric size and positive zeta potential values. The presence of the PEG chain produced a decrease in the surface charge, but the condensing ability was not negatively influenced, as shown by the ethidium bromide assay. The gene silencing capacity mediated by a siRNA-control was negligible; meanwhile, the use of a specific siRNA-Luc was able to reduce luciferase expression a 14% and 20% on average in HeLa and LS174 cells, respectively, compared to siRNA-control (*p* < 0.05). The presence of the peptides did not improve the gene silencing activity compared to plain dendriplexes. Toxicity studies confirmed the low toxicity of the PAMAM dendrimer conjugates, meanwhile LPEI polyplexes resulted to be highly toxic in the LS174T cell line.

## Figures and Tables

**Table 1:** Particle size and surface charge in the presence or absence of ligands and different N/P ratios. The data are represented as the mean ± SD (standard deviation) of three measurements.

	N/P	Size	PDI	ZP
PAMAM-G5 (PLAIN)	2	109.1 ± 0.8	0.15	32.1 ± 1.1
	4	114.7 ± 0.7	0.16	37.6 ± 0.3
	6	122.6 ± 0.9	0.17	40.0 ± 1.0
GE11	2	106.6 ± 0.3	0.09	23.0 ± 2.0
	4	105.7 ± 0.8	0.17	27.4 ± 0.5
	6	107.5 ± 1.1	0.23	27.7 ± 1.8
B6	2	71.2 ± 1.0	0.12	23.5 ± 0.9
	4	75.5 ± 0.8	0.21	20.3 ± 0.6
	6	n.d.	n.d.	n.d.
Cys	2	103.0 ± 3.0	0.24	21.1 ± 1.1
	4	125.0 ± 29.0	0.37	25.4 ± 1.1
	6	199.3 ± 19.7	0.51	26.0 ± 2.0

PAMAM: polyamidoamine, N/P: nitrogen/phosphate ratio, PDI: polydispersion index, ZP: zeta potential
